# Impact of free maternal health care policy on maternal health care utilization and perinatal mortality in Ghana: protocol design for historical cohort study

**DOI:** 10.1186/s12978-020-01011-9

**Published:** 2020-10-30

**Authors:** John Azaare, Patricia Akweongo, Genevieve Cecilia Aryeetey, Duah Dwomoh

**Affiliations:** 1 Department of Health Policy Planning and Management, University of Ghana School of Public Health, Legon, Accra, Ghana; 2 Department of Biostatistics, University of Ghana School Public Health, Legon, Accra, Ghana

**Keywords:** Stillbirth, Impact evaluation, Treatment effect, Delayed reimbursement, Mort-né, Évaluation de l’impact, Effet de traitement, Rembourcement retardé

## Abstract

**Background:**

Ghana introduced what has come to be known as the 'Free’ Maternal Health Care Policy (FMHCP) in 2008 via the free registration of pregnant women to the National Health Insurance Scheme to access healthcare free of charge. The policy targeted every pregnant woman in Ghana with a full benefits package covering comprehensive maternal healthcare.

**Purpose:**

This study seeks to measure the contribution of the FMHCP to maternal healthcare utilization; antenatal care uptake, and facility delivery and determine the utilization impact on stillbirth, perinatal, and neonatal deaths using quasi-experimental methods. The study will also contextualize the findings against funding constraints and operational bottlenecks surrounding the policy operations in the Upper East Region of Ghana.

**Methods:**

This study adopts a mixed-method design to estimate the treatment effect using variables generated from historical data of Ghana and Kenya Demographic and Health Survey data sets of 2008/2014, as treatment and comparison groups respectively. As DHS uses complex design, weighting will be applied to the data sets to cater for clustering and stratification at all stages of the analysis by setting the data in STATA and prefix Stata commands with *‘svy’*. Thus, the policy impact will be determined using quasi-experimental designs; propensity score matching, and difference-in-differences methods. Prevalence, mean difference, and test of association between outcome and exposure variables will be achieved using the Rao Scot Chi-square. Confounding variables will be adjusted for using Poisson and multiple logistics regression models. Statistical results will be reported in proportions, regression coefficient, and risk ratios. This study then employs intrinsic-case study technique to explore the current operations of the ‘free’ policy in Ghana, using qualitative methods to obtain primary data from the Upper East Region of Ghana for an in-depth analysis.

**Discussion:**

The study discussions will show the contributions of the ‘free’ policy towards maternal healthcare utilization and its performance towards stillbirth, perinatal and neonatal healthcare outcomes. The discussions will also centre on policy designs and implementation in resource constraints settings showing how SDG3 can be achievement or otherwise. Effectiveness of policy proxy and gains in the context of social health insurance within a broader concept of population health and economic burden will also be conferred.

**Protocol approval:**

This study protocol is registered for implementation by the Ghana Health Service Ethical Review Committee, number: GHS-ERC 002/04/19.

## Résumé

**Arrière-Plan:**

Le Ghana a introduit ce que nous appelons aujourd’hui Politique de Soins Médicaux Maternels ‘Gratuits’ (Free Maternal Health Policy (FMHCP)) en 2008 à travers l’inscriptions des femmes enceintes au projet national d’assurance santé afin de leur permettre d’avoir accès aux services médicaux ‘gratuits’. Selon ce projet, chaque femme enceinte au Ghana doit bénéficier d’une manière complète des services médicaux maternels.

**Objectif:**

Cette étude a pour objectif, d’évaluer la contribution de la Politique de Services Médicaux Maternels ‘Gratuits’, aux services de consultations prénatales et les accouchements à l’hôpital ainsi que déterminer l’impact de la politique sur l’enfant mort-né, périnatal et les décès néonatals en employant des interventions quasi-expérimentales. L’étude contextualisera également les résultats en tenant compte des contraintes de financement et les difficultés d’opération du projet qui portent sur le fonctionnement du projet dans la Région de Haut-Est.

**Méthodologies de recherche:**

Cette étude suit un plan de méthodologie variée afin d’évaluer l’efficacité du traitement en employant des *variables* produites à partir des données historiques du Ghana et des données démographiques de santé du Kenya(DHS) de 2008/2014, comme traitement et la comparaison des groupes, respectivement. Le DHS emploie un plan complexe, des données différentes seront évaluées afin de s’occuper des groupes divers et aux stratifications à tous les niveaux de l’analyse en mettant les données en STATA et préfixer les commandes du Stata avec ‘svy’.

Ainsi, l’impact du projet sera évalué en utilisant des plans quasi-expérimentaux, la mise des scores dans le bon endroit, et la différenciation de méthode, la prédominance, la différence dans les moyennes et le test d’association entre le résultat final et les divergences dans les résultats seront tous obtenus en utilisant le *Rao Scot Chi-square*. Les variables déconcertantes seront ajustées après avoir employé Poisson et multiples modèles de régression logistiques. Les résultats obtenus sous forme de compte rendu statistiques seront présentés en pourcentages, en coefficients régressifs et en proportion de risques.

Cette étude emploie, donc, la technique d’étude de cas intrinsèques afin d’explorer les opérations courantes du Projet ‘gratuit’ au Ghana en utilisant des méthodes qualitatives afin de produire des données primaires dans la Région du Haut-Est. Pour une analyse approfondie.

**Discussions:**

L’étude révèlera les contributions du Projet aux services médicaux maternels et sa performance en ce qui concerne l’élimination du phénomène des enfants mort-nés, périnatals et les résultats de services médicaux maternels et néonatals. Les discussions se concentreront sur le plan du projet et sa réalisation tout en tenant compte des contraintes de ressources en montrant si l’Objectif de Développement Durable (Objectif 3) sera atteint ou non. L’efficacité du Projet ainsi que ses succès en ce qui concerne l’assurance santé sociale dans le contexte de la santé publique et les difficultés économiques seront également discutées.

**Approbation protocolaire:**

Cette étude a été approuvée pour mise en exécution par le Comité de Vérification Ethique du Service de la santé publique du Ghana: GHS-ERC 002/04/19.

## Plain language summary

In July 2008, Ghana introduced what is termed the Free Maternal Health Care Policy across the country through the free registration of all pregnant women to its National Health Insurance Scheme (NHIS). The policy objective was to eliminate the financial barrier to access thereby increasing antenatal care up-take and facility delivery utilization among pregnant women and postnatal care service for nursing mothers with their newborn babies, free of charge up to 90 days.

The objective of this current study is to determine the magnitude of utilization the policy chalked since its inception and to measure its effect on newborn care outcomes in Ghana. The study also wants to examine the policy used in its current state, against the background of delayed claims payments by the national health insurance scheme to service providers.

This research is in two parts: first, using existing data from Ghana and Kenya, and comparing between the two countries; Ghana as the group that received the ‘free’ policy and Kenya as the group that did not receive the policy at the time. The second part of the study will collect information from doctors, midwives, hospital administrators and pregnant women in the Upper East Region of Ghana, one of the regions with a high subscription to the ‘free’ policy and also examined available records in the hospitals, to understand and explain the policy current state and how that clarifies the results from the existing data.

This study is expected to contribute to knowledge in the area of the policy implications for newborn care in Ghana. Policy gaps will be thoroughly discussed, as it pertains to scarce resource and funding difficulties in developing countries in Sub-Saharan Africa. The study will measure in-country strides i.e. Ghana’s feat towards the attainment of Sustainable Development Goal 3 and make projections and predictions on Ghana’s chance of realizing the 12 per 1000 live births rate set for countries by the World Health Organization (WHO) to be achieved by the year 2030.

The study will offer rich literature to research and policy in the area of newborn care and outcomes, a rather understudied concept, in Ghana and Sub-Saharan Africa.

## Background

Despite the modest gains from maternal and child healthcare interventions across the world, reproductive health issues remain a global challenge, particularly in developing countries. It is estimated globally that, more than 300,000 women died out of pregnancy or childbearing related conditions in 2015, with about 3 million neonatal deaths and 2.6 million stillbirths [1–4].

Among these figures exist a sharp disparity between and within countries. For example, stillbirth rates in Japan reduced from 18 per 1000 live births to 3 per 1000 live births between 1979 and 2010. In the United Kingdom, it was estimated that 1 in 200 pregnancies end in stillbirth [5–7].

Conversely, Nigeria alone, Africa’s most populous country recorded 42 per 1000 live births in 2015, which translated to over 300,000 babies dying before they are born [20, 21]. Albeit Ghana compares better to Nigeria, 28 per 1000 live births were also reported about the same period [8, 9]. In general, more than 98% of the world stillbirth estimates are accounted for by developing countries, particularly Sub-Saharan Africa and South Asia [10–13].

Recent studies have shown that quality antenatal care, during which time screenings are carried out on pregnant mothers to pick high-risk ones for target monitoring and management, can improve pregnancy outcomes and also minimize the risk of maternal morbidity and mortality [3, 14–16]. This principle holds for skilled delivery as well, often at a healthcare facility, where high-risk mothers and babies are managed using structured programs of emergency obstetric and neonatal intensive care [17, 18].

Nonetheless, country level and household income disparities exist widely across regions and have rather restricted access to maternal healthcare to the privileged, thereby stretching the inequality margin. Consequently, political leaders have introduced pro-poor policies as the panacea to bridging the access gap relative to maternal and child healthcare [19–22].

Within country policy interventions have ranged from equitable access to healthcare to the provision of quality healthcare at all levels for pregnant mothers and newborns [22–24]. In a similar vein, Ghana introduced one of such policies in 2008 dubbed, the ‘Free’ Maternal Healthcare Policy (FMHCP) with a primary aim of increasing facility delivery utilization and to achieve maternal and newborn care in general [3, 25, 26].

Essentially, the ‘free’ policy lacked an operational framework for implementation, but had a political directive that ensured that every pregnant woman gets to register with the national health insurance scheme (NHIS), without paying a premium, and then access comprehensive maternal healthcare free of charge from NHIS accredited health service providers up to 90 days post-delivery [25, 27].

The government of Ghana has since invested several millions of Ghana cedis in a bid to keep the policy running amidst NHIS reimbursement challenges. With nearly 3million women benefitting from the ‘free’ package as by 2013 [28, 29], studies are yet to report on the policy gains or otherwise relative to perinatal healthcare outcome.

Despite the huge financial investment on the free policy intervention, literature is rather restricted to reporting on a nominal increase in attendance at healthcare facilities regardless of time-variant factors and somewhat monotonously on factors that affect the implementation process. Moreover, the existing literature turns to focus more on the policy effects on mothers and less on the unborn and the newborn.

Thus far, the ‘free’ maternal health care policy relationship with maternal healthcare utilization and outcomes, vis a vis medium to long term impact on perinatal healthcare has yet to be adequately documented [30–32].

Incidentally, perinatal death is also poorly researched in Ghana and Sub-Saharan Africa despite its economic burdening abilities [11, 14]. Stillbirth which accounts for a large portion of perinatal deaths has been estimated to have dire consequences on household healthcare expenditure, particularly in areas where the ratios are considered worse than child mortality due to malaria and HIV/AIDS [33–35].

This notwithstanding, perinatal deaths continue to receive little attention in public health and policy research [36, 37]. Hence, this current study seeks to examine the ‘free’ policy effect on maternal healthcare utilization after a decade of implementation and to measures its contributions towards the reduction of stillbirth, perinatal deaths, and neonatal mortality in Ghana.

## Overall study goal

To evaluate the contribution of the ‘free’ maternal health care policy towards maternal healthcare utilization and measure its impact on perinatal healthcare outcomes in Ghana.

## Specific objectives

1. To determine any significance in association between the FMHCP and antenatal care uptake, and facility delivery utilization in Ghana.
2. To compare the difference in perinatal outcomes between Ghana and Kenya relative to the ‘free’ maternal health care policy.
3. To measure the treatment effect of facility delivery on stillbirth, perinatal deaths, and neonatal mortality among women who received the FMHCP program compared to those who did not receive the program.
4. To describe the ‘free’ maternal health care policy implementation in its current state in Ghana, within the context of funding constraints and implementation bottlenecks.

## Research questions

The study will address the following research questions

1. What is the relationship between antenatal care, facility delivery utilization, and the free maternal health care policy?
2. What is the treatment effect of facility delivery utilization on stillbirth among women who received the ‘free’ maternal health care policy?
3. What is the treatment difference of facility delivery utilization on perinatal and neonatal mortalities among women who received the ‘free’ policy treatment, compared with those who did not receive the policy?
4. How free is the ‘free’ maternal health care policy relative to implementation, against the background of delays in claims paid by the NHIS in Ghana?

## Hypothesis

This study hypothesis that;

1. H_0_:There is no significant association between antenatal care uptake and the free maternal health care policy.
2. H_0_:There is no significant association between facility delivery utilization and perinatal deaths among women who received the free maternal healthcare policy.
3. H_0_**:**Facility delivery utilization has no significant treatment effect on perinatal deaths among women who received the free maternal health care policy.

## Conceptualization

Literature has shown that drivers of maternal and child healthcare include characteristics such as; maternal age, gestational age, parity, educational level, wealth index or income, multiple pregnancies, and area of residence [10, 38–40]. These factors are thought to significantly influence the uptake of antenatal care and skilled delivery [41–43]. The overall effect of the factors are moderating and have direct or indirect links to undesirable perinatal outcomes.

Institutional level factors also exist, often at the point of service delivery, and tacitly restrict the full realization of healthcare objectives. They impede policy implementation and influence outcomes of health policy interventions. For example, factors such as staff competence, health facility location, and quality of care are critical to the attainment of the primary outcomes of interest and also play roles to influence the undesirable outcomes.

This study attempts to draw lessons from the Street-Level Bureaucratic theory, as propounded by Lipsky, to examine how service providers, the lower level employee negotiate with policy users to partner government in the implementation of the FMHCP for optimum effect.

Lipsky opined that in policy implementation, the lower level employee is faced with challenges and exercises discretions. He maintained that the employee is the last link to policy formulation and should not be left out in developing workable policies [44, 45] and that, the lower-level employee which he termed the Street-Level Bureaucrat, is the one with the actual implementation role of public policy [48, 46].

**Fig. 1:**
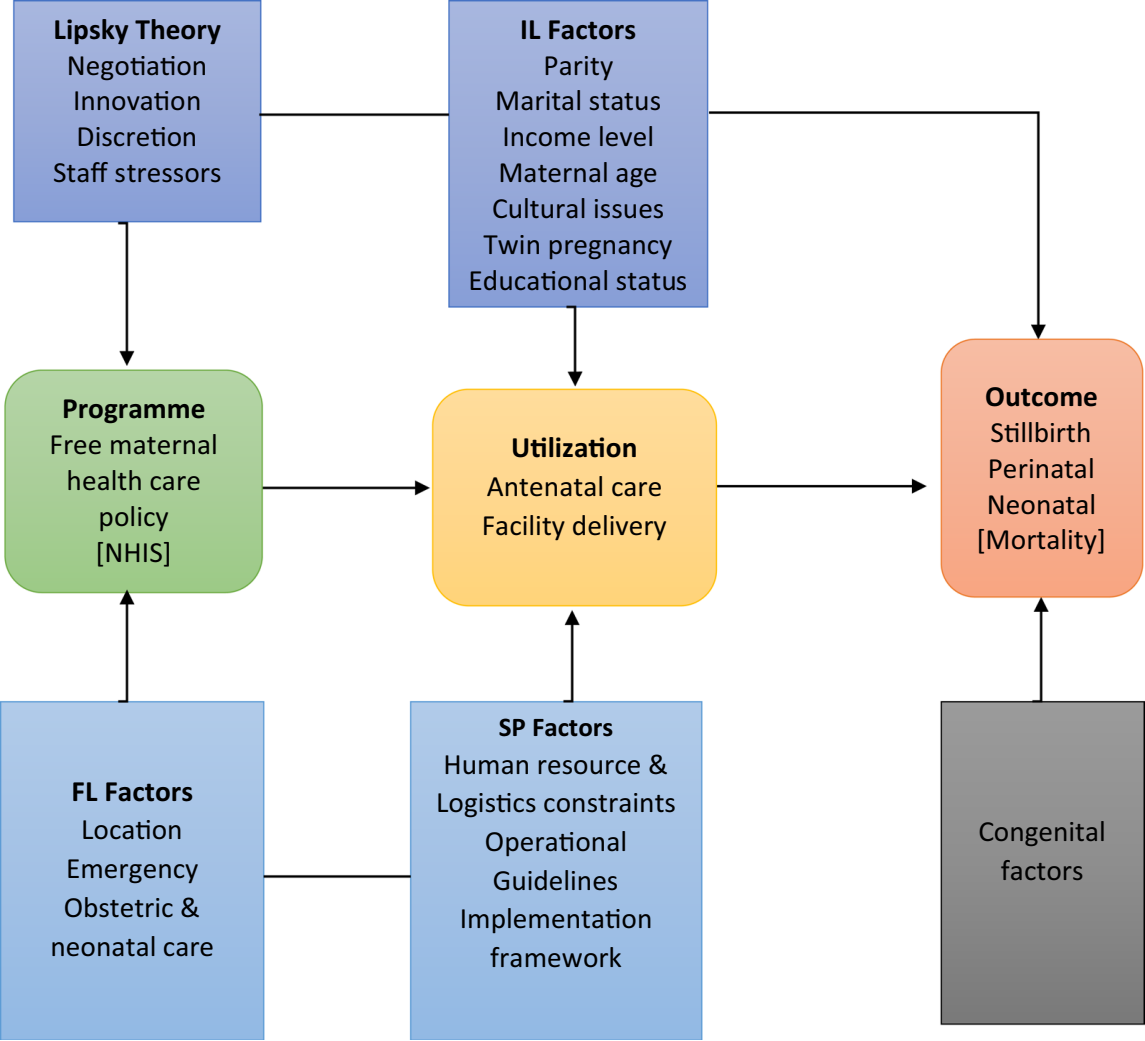
Conceptual framework showing causal relationship between actors and outcome

The ‘free’ maternal health care policy in practice exercises causal effects through policy effectiveness, and therefore undesirable effects if challenges are present. Thus, this current study proposes a framework (Fig. 1) of quantitative and qualitative design to examine the FMHCP quantum contribution towards maternal healthcare utilization and its effects in combating perinatal deaths.

Also, the study examines the policy, as being implemented, adopting Lipsky’s idea of street-level bureaucracy to addressing a critical question; to what extent does the lower level employee influences the successes of the ‘free’ maternal health care policy in Ghana against the backdrop of funding constraints and implementation challenges.

## Methodological literature

Studies on perinatal health care outcomes in Ghana are scanty and restricted. Existing studies often bothered on stillbirths reported within districts and institutions [20, 21, 45]. Despite the contributions of earlier studies to literature, their findings are limited in scope and do not allow for conclusion relative to in-country strides towards the achievement of the sustainable development goal 3.

The limited research on policy impact on neonatal death highlights the challenges of inadequate research in Ghana context. Of critical importance to policy, continuity is the need to assess policy gains overtime after policy implementation [48, 46].

The one hunch of the ‘free’ policy in its prospect, was to bridge the financial gap to maternal healthcare access and create a situation of increased utilization in maternal healthcare. Nevertheless, copious literature exists to suggest that service providers are rather owed huge sums of monies, a situation which leads to service provider ineffectiveness due to acute shortages of supplies and consumables [43, 47, 51].

This current study aims to measure the impact of the ‘free’ maternal healthcare policy; firstly, on maternal healthcare utilization and secondly, on the effect of facility utilization on stillbirth, perinatal death, and neonatal mortality.

Specifically, the study adopts quasi-experimental methods of propensity score matching technique and difference-in-differences analysis to determine the ‘free’ policy contributions towards the uptake of antenatal care and facility delivery utilization, and its impact in reducing stillbirth, perinatal deaths, and neonatal mortality.

As a novelty, this study also collects qualitative data in an intrinsic case study style, using in-depth interviews and focus group discussions (FGD) to explain the context within which the so-called ‘free’ policy operates.

Impact evaluation design is broadly categorized into two; prospective evaluation design and retrospective evaluation design. The former is designed during the program design stage and incorporated into the implementation plan, in which case, baseline data is collected at the pre-implementation stage using pre-defined variables of interest. Treatment assignment in prospective designs has the advantage of randomization, which is the gold standard.

However, not all programs have the benefits of randomization, particularly, public health programs which are usually targeting populations such as poor communities and vulnerable groups. In these situations, it becomes crucial to adopt quasi-experimental design techniques in evaluating the program in the absence of randomization [49–58]. This is referred to as retrospective design, and is the method of choice for this study, giving the social policy status of the ‘free’ maternal health care policy.

Retrospective design is usually the option available when impact evaluation was not envisaged and incorporated in a public program at the design stage, and in this case, statistical techniques are used to generate the propensity score of treated and untreated units’ characteristics for comparison to determine the treatment difference [49, 51, 52].

## The free maternal healthcare policy in Ghana

The FMHCP was introduced in Ghana through a political declaration in July 2008 by the government of Ghana, with no prior policy framework. Thus, the Ministry of Health (MOH) was tasked to ensure the successful implementation of the policy through the NHIS, an agency under the MOH. In principle, the MOH was tasked to enable the free registration of clinically confirmed pregnant women to access care from NHIS accredited facilities without paying out of pocket.

The policy had no sustainable funding source at the time but relied on an initial £42.5 million ($54.2 million) pledge from the UK government [53]. No clear cut eligibility criteria existed. One needed to be confirmed pregnant by a registered nurse, midwife, or doctor to be enrolled in the national health insurance scheme (NHIS) in the nearest office.

Between 2008 and 2014, the police had registered around 3 million women who benefited from comprehensive maternal healthcare in Ghana at no cost to them, but to the NHIS [28, 54]. With no premium payment, the NHIS appears short-circuited in terms of funding and perhaps, would have to rely on other sources, conventional or otherwise, to fund the ‘free’ policy.

Unsurprisingly, the insurance scheme has since reported funding deficit since the policy implementation characterized by erratic payment of service provider claims, which is copiously documented [47, 55–57]. The slow pace of reimbursement coupled with NHIS card issuance difficulties has created a situation of uncertainty surrounding the implementation of the ‘free’ policy [58].

**Fig. 2:**
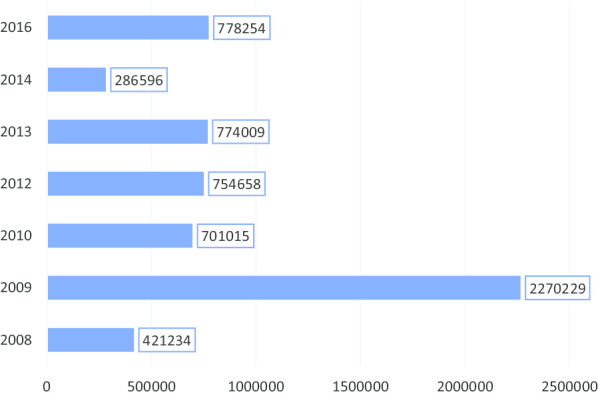
Trend of free maternal health care policy enrollment

It is in this light that, this study will seek to explore the current situation of the policy implementation by collecting in-depth primary data from the field of implementation i.e. hospital, clinic, and the policy users to situate the discussions of this study into proper context. Figure 2 presents the number of pregnant women beneficiaries of the policy, while Table 1, below show the proportional changes over the years, indicating inconsistencies in the trend.

**Table 1:** Registration under the free maternal health care

Year	No. of enrollment	Percentage change
2008/2009	383,216	–
2010	504,609	24%
2011	485,460	− 4%
2012	754,658	36%
2013	774,009	3%
Total	2,901,952	

Source: NHIA Report, 2013

## Operational definitions

*Utilization*: The use of maternal healthcare services rendered in a healthcare facility at any level.

*Maternal healthcare*: Antenatal care clinics and facility delivery services provided to pregnant women in a healthcare facility.

*Antenatal care uptake*: This refers to a 4+ antenatal care visit, thus, antenatal clinic visits of less than 4 are equal to zero visits (or 'no visit').

*Facility delivery*: This is when a pregnant woman gives birth in a healthcare facility at any level.

*Stillbirth*: This refers to the birth of a fetus with no signs of life, after 28 weeks of gestation or the death of a fetus within 24 h after birth.

*Perinatal death*: This refers to the number of stillbirth plus the death of neonates within the first week of life.

*Neonatal deaths*: This refers to the number of newborn deaths within 28 days of life.

*Treatment group*: Pregnant women study participants registered with the national health insurance scheme and made 4+ antenatal care visits or pregnant women participants in Ghana who delivered in a healthcare facility.

*Comparison group*: Pregnant women study participants registered with the national health insurance but have made less than 4 antenatal care visits with less than four minimum antenatal visits or pregnant women who did not deliver in a healthcare facility within the study period.

## Methodology

### Study design

This study employs a mixed methodology in two-prongs. The first prong uses two quasi-experimental methods of a difference-in-differences (DID) analysis and propensity score matching (PSM) technique to analyze Demographic and Health Survey data sets of Ghana 2008/2014 and Kenya 2008/2014. Thus, the 2008 data set for the two countries forms the baseline data, while, the 2014 DHS data sets of countries, represents the end line data set.

The DID method necessarily requires comparable data sets from a different country, other than Ghana, that had not implemented the intervention (the 'free' policy) at the time, hence Kenya. The rationale for choosing Kenya is further explained below and will allow for comparison using data from a non-intervention country at the time, but with similar timelines data sets from DHS. This is also necessary for testing the parallel trend assumption applied in DID.

The propensity score matching (PSM) technique will also be used to estimate the average treatment effect on the treated through the generation of the propensity scores of the two groups; the treated group, post-intervention (2014 DHS) and the untreated group, pre-intervention data (2008 DHS) for comparison using the observed characteristics. The study then assumes that the unobserved characteristics are similar and cancels out, applicable in PSM.

The second prong of the study employs an intrinsic care study design to effect, collecting primary data through one-on-one in-depth interviews, and focus group discussion techniques from the Upper East Region of Ghana for analysis. Inductive thematic analysis will guide the management of the qualitative data using INVIVO 12, with vivid quotations to convey participants’ impressions.

The mixed-method design is driven by the overarching goal of the study; first to measure the free maternal healthcare policy effect on maternal healthcare utilization and to determine the impact of the utilization on stillbirth, perinatal death, and neonatal mortality.

**Fig. 3:**
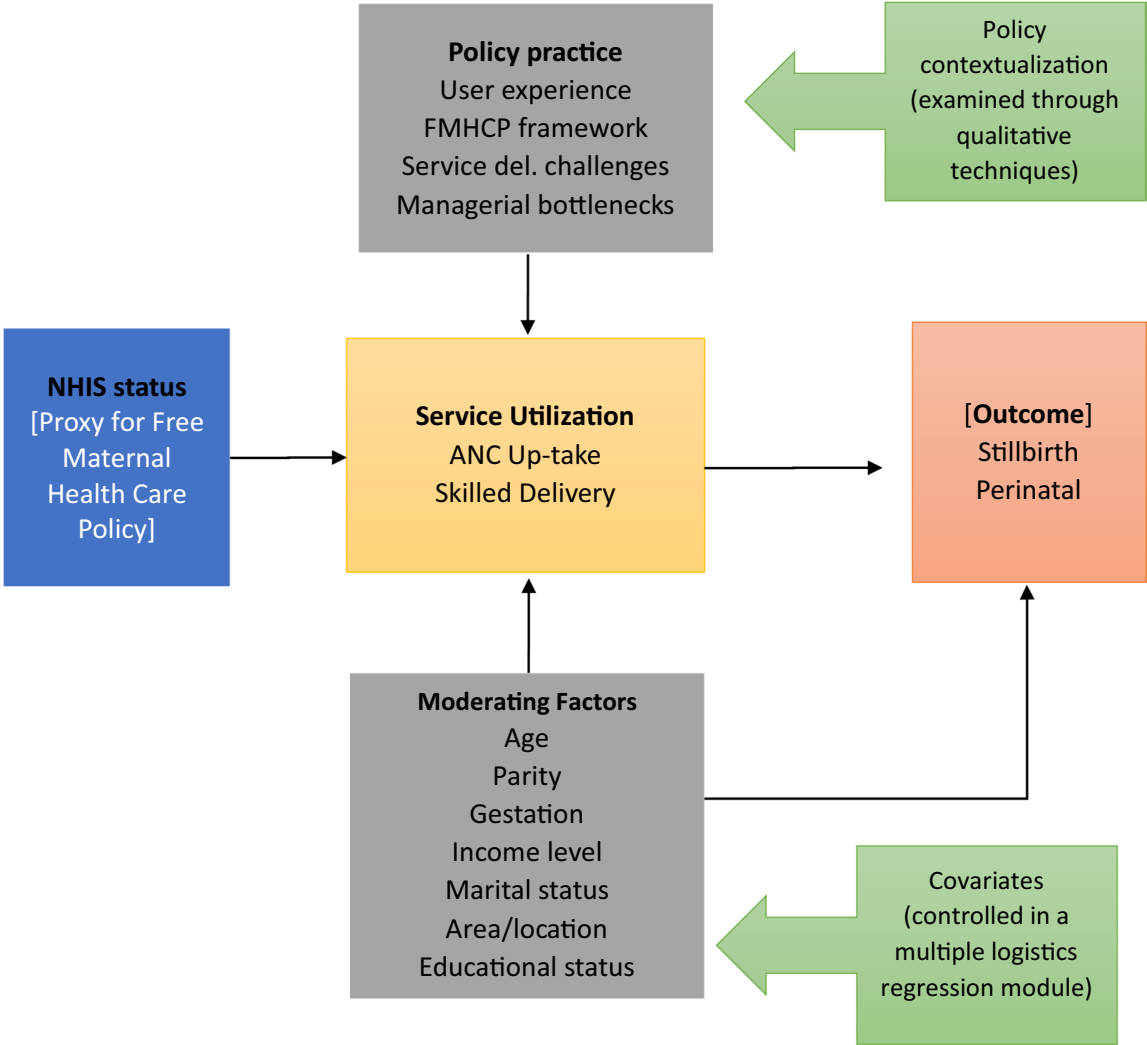
Study design showing related variables and confounders

Secondly, the study aims to explore an in-depth understanding of the policy in practice under its current operational constraints to explain policy workability from user and service provider perspectives. Figure 3, presents the diagrammatical representation of the study design.

### Kenya as a comparison country

Comparatively, Kenya showed relevant commonalities as Ghana and was chosen as a comparison country. First, Kenya DHS data were collected about the same time as Ghana and had no such intervention within the time frame. This is necessary for the DID analysis.

Secondly, the DHS reports of the two countries show similarities of related variables of interest in this study. For example, Ghana recorded 30 and 50 per 1000 live births for neonatal and infant mortality rates respectively, and within the same period, Kenya also recorded 31 and 52 per 1000 live births for neonatal and infant mortality, according to their respective 2008 DHS reports [59–61].

Also, Kenya was considered appropriate giving its geopolitical and socioeconomic similarities to Ghana such as; political stability, literacy rate, lingua franca, and Sub-Saharan location.

### Data source and size

Ghana and Kenya 2008/2014 DHS data sets are collected about the same time by MEASURE DHS, a USAID sponsored program. DHS used a similar questionnaire of international standards to collect the 2008/2014 data sets, which serves as the main data source for this study. The advantage of using DHS data has been widely documented [62, 63]. DHS collects wide and multipurpose data with a standard questionnaire which includes health indices and contains variables relevant for this current study.

Children data sets, KR data, collected data from women in household settings within their reproductive ages of 15–49 years of both countries will be used for the quantitative analysis. Cross country data analysis will only be required at the stage of the quantitative analysis owing to the DID method.

However, primary data will be collected from one selected region in Ghana, the Upper East Region, based on the odds of coverage of the ‘free’ policy, for an in-depth description of the policy in the real-world setting. This is relevant to add context to the study findings and empower the discussion. Ghana’s data sets are represented by the 2008/2014 GDHS data collected from 12,323/12,831 households respectively using a two-stage sampling technique.

Kenya, on the other hand, is represented by the KDHS data sets of 2008/2014, also collected using two-stage sampling design from a representative household sample of 9936/40,300 from 400 clusters (133 urban, 267 rural) and 1612 clusters (997 rural, and 617 urban) for the 2008/2014 respectively [45, 48].

In total, the sample size of Ghana 2008/2014 DHS data are n = 2992/5884, whereas, those of Kenya are n = 6079/20,964 for the 2008/2014 respectively. The two countries' data sets were collected, approximately 6 years into the implementation of the ‘free’ maternal healthcare policy in Ghana, and considered adequate and representative in size to power the study.

### Sample weighting

Due to the complex nature of the two-stage sampling design, which essentially produces a complex survey data sets, sample weighting will be applied across the data sets, to cater for clustering (the enumeration areas) and stratification (urban/rural area of residence) for precision. Sample weighting will be set in STATA 15 software, using variable *v021* and v022 from the data sets, as primary sampling units and strata respectively. Thereafter, all commands will prefix the svy (survey) to apply sample weighting in the analysis*.*

The choice of rural/urban area of residence allows for socio-economic variation to be incorporated into the data composition and analysis. Hence, robust standard error (Taylor Linearization and bootstrapping) approach will be used to adjust for clustering and stratification bias.

Sample probability weighting will be generated through the selection of sample weighting variable *v005* from the data sets and divided by 1000,000, to cater for the 6 decimal places, usually not reported in Demographic and Health Survey data, thus, stata command of;

*gen wgt* = *v005/100,000,* then, *svyset [pw* = *wgt], psu (v021) strata (v022) to set the DHS data.*

### Statistical methods and rationale

The design and intentions of the ‘free’ policy could not allow for randomization, the gold standard for impact evaluation, hence, quasi-experimental methods; DID and PSM techniques are adopted for the analysis of the secondary data sets. These methods are considered appropriate in evaluating public health programs that have no randomization in the policy design and implementation stages, and in particular, suitable for policies whose choice of participation is somewhat open and voluntary.

Combining the two methods gives the advantage of one making up for the limitations posed by the other. The DID analysis will employ the 2008 data sets of the two countries as baseline data (the starting time), whereas the 2014 DHS data sets of the same will represent the end line (the end time), as shown in Table 2.

**Table 2:** Difference-in-differences method

	Before treatment	After treatment	Difference
Treatment (T)	X_0_	Y_0_	T = Y_0_–X_0_
Comparison (C)	X_1_	Y_1_	C = Y_1_–X_1_
Difference	X_0_–X_1_	Y_0_–Y_1_	DD = (Y_0_–X_0_) – (Y_1_–X_1_) = T-C

The choice of propensity score matching technique is also appropriate for the estimation of the policy average treatment effect through the generation of comparators ex-post. A drawback of PSM is the risk of lack of common support for paring treated units to the untreated. This particularly occurs when the sample size is small, so-called the curse of dimensionality. However, this study will merge the 2008/2014 data sets to increase the sample size and to minimize the risk of the curse of dimensionality [49].

Increased sample size will also enhance the study power for precision, albeit, also determined in the original two-stage DHS sampling design. The combined effect of the two quasi-techniques will eliminate the limitation of one and enhance the robustness of the study results [64, 65].

From Table 2 above, before treatment (baseline) is represented be GDHS 2008 (X_0_) for the treatment, T, and KDHS 2008 (X_1_) for the comparison group, C. The after treatment (end line) is also represented by Y_0_ and Y_1_ for Ghana DHS 2014 and Kenya DHS 2014, respectively. Thus, the DID analysis is the difference of T = (y_0_–x_0_) and C = (y_1_–x_1_); estimate for the before treatment difference and the after-treatment difference. The differences between the two represent the treatment effect, thus, the treatment effect is = (y_0_–*x*_*0*_) − (y_1_–*x*_1_), which is equal to T-C.

### Demographic and health survey data

Demographic and Health Survey data sets are a household survey collected using a two-stage sampling method. Clusters for the sampling in Ghana used sampling frames of the 2000 and 2010 population and housing census to sample for the 2008 and 2014 DHS surveys respectively. For 2008, 412 clusters were determined in the first stage. In the second stage, 29 households were selected from each of the 412 clusters. Household from one cluster data was left out due to ethnic conflict at the time, thus 12, 323 households were selected.

The 2014 DHS also determined 427 clusters; 216 urban areas and 211 rural areas. The second stage then sampled 30 households per cluster for questionnaire administration in each household, thus, 12,831 households, similar to the sampling technique for the 2008 DHS.

In both situations, women between the ages of 15 and 49, who were either permanent residents in the selected households or stayed in the household the night before the selection were eligible to participate in the study and had their blood pressure measured. Sampling excluded women living in barracks, hotels, and prisons. Children between 0 and 59 months were also eligible and had their height and weight checked.

Eligible women participated in the study and answered questions on; age, pregnancy status, education, and relationship to the household head, residence, birth history, parity, pregnancy outcomes, knowledge and use of family planning methods, malaria interventions, diarrheal diseases, antenatal care and facility, and facility delivery (children born within the last 5 years).

Whereas GDHS 2008 collected data on the NHIS, the GDHS 2014 collected information on the free maternal health policy. Ghana and Kenya DHS surveys are similar in sampling procedures and methods and therefore comparable. DHS surveys are considered alternative to population and housing census in many situations due to their wide range of coverage in public health and socio-economic variables.

### Qualitative data setting

The second prong of the study employs a qualitative design to collect data from the Upper East Region (UER) of Ghana for analysis. The region is one of the poorest regions of Ghana [58, 66]. It is located in the north-eastern part of Ghana, bounded to Togo at the east, to Burkina Faso at the north, to the Sissala District at the west and the South by the West Mamprusi District.

The region is located between longitude 0° and 1° West and latitude 10° 30′ N and 11° N and has a total land area of about 8842 sq km with Bolgatanga as its capital. According to the 2010 population and housing census the population of the Upper East Region is estimated to be around 1,046,545 people, with 540,140 women, representing 51.6% of the total population [67].

The region has a national health insurance enrollment rate of 6.3% [68]. The majority of health care facilities in the region are publicly owned, with a good number of midwives comparatively. The Upper Region is a key player in the area of maternal healthcare utilization and has been a leader in the regional league since 2012 in the area of maternal healthcare.

According to the 2013 National Health Insurance Authority (NHIA) report, there are 211 accredited NHIS service providers in the region, many of which are clinics and health centers [51]. Ethnic groupings are sparsely distributed across the capital town; Bolgatanga at the center, Bawku area (to the east of the capital), and Navrongo area (to the west of the capital).

### Study population and sampling

Primary data will be collected from doctors, midwives, hospital administrators, and pregnant women in the Upper East Region. The region grouped into three zones; Central, Eastern, and Western Zones. One district hospital and two health centers each will be selected from the three zones using a simple random sampling technique, from where study participants will be recruited for the qualitative data collection.

Doctors from O&G departments in the hospitals, midwives from the labor ward and antenatal clinics in hospitals and health centers/CHPS compounds, and hospital administrators will be recruited purposively for one-on-one interviews. Even though hospital settings will be ideal to explore policy implementation bottlenecks, the inclusion of health centers will give rural perspectives to policy implementation from a typically developing country setting.

Pregnant women attending antenatal care (ANC) clinics in the selected facilities will also be recruited using convenient sampling methods after explaining the study rational to them at the antenatal clinic schools. The inclusion of pregnant women will add policy-user experience to the service provider perspective, epitome for the understanding of multiple realities as espoused by Creswell [69].

The Regional Director of Health Service for Upper East Region and the Director for Policy Planning Monitoring and Evaluation of the Ghana Health Service Headquarters will also be interviewed as Key Informants to provide top-level managerial context and viewpoints. Their inclusion will enhance data triangulation from multiple sources i.e. policy users, services provider perspective, and policy implementers viewpoint. The director's opinion will also serve to validate the results from the provider perspectives.

### Data collection

An open-ended interview guide will be used for the in-depth interviews (IDI), while fictional cases, developed for the purpose will guide the Focus Group Discussion (FGD). Interview sessions will be one-on-one and will last between 1 and 2 h per participant. Follow up questioning will be used to obtain clarity, where necessary.

The advantage of FGD is that it eliminates the fear of tagging and potential victimization among study participants while generating an in-depth understanding of participants' experience as Patton puts it "qualitative method permits inquiry into the selected issue in great depth with careful attention to detail, context, and nuance…”[70, 71].

**Fig. 4:**
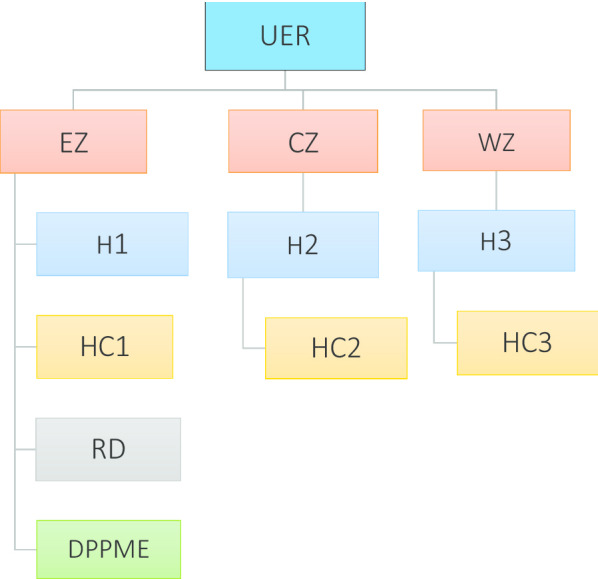
Qualitative data collection tree showing source of primary data

The in-depth interviews and the group discussions will be audio tape-recorded and later transcribed verbatim for the analysis. The rationale for audio recording is to keep track of every comment shared by participants and this will be explained to participants to gain their consent. Table 3, below is a summary of the sample size, while Fig. 4 shows the clustered units of facilities, from where samples will be drawn.

**Table 3:** Target participants

Method	Target	Size	Duration
KII	Director, PPME	1	1 h
KII	Regional director	1	1 h
IDI	Doctors	3	3 h
IDI	Midwives	12	6 h
IDI	Administrators	3	3 h
FGD	Pregnant women	30	3 h
Total		50	17 h

### Inclusion criteria

Only staff (doctors, midwives, and administrators) of the selected hospitals, clinics, health centers, and CHPS compound with a minimum of three (3) years of working experience in the labor ward and antenatal clinic or facility will be interviewed. Also, only pregnant women of at least 18 years who access antenatal care or delivery services using national health insurance cards will be included in the focus group discussion.

### Exclusion criteria

Pregnant women whose health status is deemed unstable, using a parameter of vital signs; blood pressure, pulse, and respiration by the American Heart Association as shown in Table 4, will be excluded from the study. This is in line with the principle of non-malfeasance.

**Table 4:** Vital signs criteria

Vital signs	Lower limit	Range	Upper limit
Blood pressure	90/60	90–130/60–85	130/85
Pulse	70	70–100	100
Respiration	16 cpm	16–22 cpm	22 cpm

### Statistical assumptions

The parallel trend assumption guide the use of DID analysis and assumes that no time-varying differences exist between the treatment and comparison groups of both countries. The second critical assumption for the difference in differences method is that the outcomes of interest in the two countries will move in tandem with or without the treatment. The Propensity Score Matching technique on the other hand assumes that, no systematic differences exist in the unobserved characteristics between the treatment group and the comparison group.

### Variables of interest

The maternal healthcare utilization examines two primary outcome variables which include; antenatal care up-take (4+ visit = 1, and ≤ 3 visits = 0), and facility delivery utilization, also binary outcome (facility delivery = 1 and home delivery = 0). Mothers’ health insurance status (proxy to the FMHCP) is the exposure variable of interest in this study.

Stillbirth, perinatal death, and neonatal mortality are secondary outcome variables of interest generated as binary outcomes from the DHS data sets using variable *b6,* and *b7* against which the policy utilization impact will be measured. Table 5 presents the derived variables generated using STATA 15.0 from the 2008/2014 DHS data sets.

**Table 5:** Variables of interest

Item	Label	Name	Recoding	Binary V
1	Respondent holds valid NHIS card	*S1015*	Holds a valid NHIS card (with or with no evidence) = 1 No valid NHIS card = 0	*Vid_1*
2	Respondent hold valid NHIS card	*S1015*	Holds a valid NHIS card (with evidence) = 1 No valid NHIS card plus (holds a valid NHIS card with no evidence) = 0	*Vid_2*
3	Number of ANC visit during pregnancy	*m14*	Antenatal visits 0–3 = 0 Antenatal visits ≥ 4 = 1	*anc4*
4		*m15*	Home delivery = 0 All facility delivery = 1	*fdelivery*
5	Age at death	*b6*	Alive after day 0 = 0 Born dead/died on day 0 = 1	*stillbirth*
6	Age at death	*b6*	Did not die before 7th day = 0 Died before 7th day of life = 1	*prdeaths*
7	Age at death (months imputed)	*b7*	Did not die before 28th day = 0 Died before 28th day of life = 1	*neodeaths*

### Exposure variables

The National Health Insurance status [proxy to the ‘free’ maternal healthcare policy (FMHCP)] is the main exposure variable. However, on the secondary level of analysis, antenatal care up-take (binary) and facility delivery utilization (also binary) are independent predictors, through which the secondary outcomes of interest; stillbirth, perinatal death, and neonatal death can be measured.

### Outcome variables

Antenatal care uptake and facility delivery utilization are the primary outcomes of interest. However, on the secondary level of analysis for the FMHCP impact, stillbirth, perinatal death, and neonatal mortality are the secondary outcomes variables, to determine the policy effect.

### Covariates

Covariates are the moderating factors identified in the study conceptual framework. Covariates that show significant association with the variables of interest under consideration will be controlled for using multiple logistic regression. These include maternal characteristics such as; age, parity, pregnancy status, cesarean section, educational status, twin pregnancy, income level, abortion, and marital status. These will be adjusted for as confounding using logistics regression models. Population characteristics such as wealth index, distance, region, educational status, and rural/urban residence will also be adjusted for as confounding variables [42].

### Scope and limitations

This study is limited in scope, as it will not estimate the free policy impact on maternal mortality. Even though the gold standard for policy impact evaluation is randomization, the FMHCP policy implementation was without randomization due to its characteristic; a social policy targeting everyone, particularly, the vulnerable and less privileged, thus, the strength of randomization as contained in a prospective design is a limitation in this design.

Again, Yin averred that the essence of a qualitative study is to generate a deeper understanding of a phenomenon rather than to generalize and that, any generalization should be done with caution and only in settings of similar characteristics [71]. Yin observation is applicable in the context of the qualitative design.

### Ethical considerations

This protocol received approval from the Ghana Health Service Ethics Review Committee (Reg. No. GHS-ERC: 002/04/19). Data access has also been granted by MEASURE DHS, after completing registration. Access to Ghana and Kenya Demographic and Health Survey data sets for the study period is approved (letter attached).

Data handling comply with the GHS-ERC protocol requirement and the ethical standards of MEASURE DHS, with no third party use. Data will be used only for the project and under the approved project title and regulations of the University of Ghana Public Health School. DHS data sets are anonymous, thus, there will be no attempts to identify communities, households, or individuals who participated in the respective surveys.

Terms of data use are referenced at https://dhsprogram.com/Data/terms-of-use.cfm. Where necessary, an electronic PDF copy of any publications from this study will be sent to archive@dhsprogram.com. All study participants will sign a consent form before participating in the interviews and group discussions.

Pregnant women participants will have their vital signs checked and recorded by a registered nurse, to ensure their health status meets a set of healthy criteria before they can participate in the FGD (Table 4). Participants’ identity will be protected and voice recording immediately transcribed into hard copies and the content discarded. All study participants will receive a Participants Information Sheet certified by the GHS-ERC.

Study participants will also have the right to withdraw from the study before or during the data collection process with no prior notice. The GHS-ERC approval letter together with an official permit from the University of Ghana Public Health School will be used to gain entrée to all primary data collection points for the qualitative data gathering.

### Protocol implementation

This protocol will be implemented in phases as per the design. Proposal writing preceded the protocol’s approval and the approved protocol will be implemented as outlined in Table 6 (see Additional file 1). The implementation will also be monitored by the Ghana Health Service Ethics Committee, as well as the supervisory committee of the University of Ghana Public Health School. The approved protocol follows the standard of research required for the award of a doctoral degree set out by the University of Ghana.

**Table 6:** Protocol implementation, monitoring & evaluation

Phase I	Phase II	Phase III
*Review of relevant literature on;* Maternal health care policy Maternal health care utilization Burden of stillbirths Perinatal deaths Neonatal mortality *Retrieval of secondary data from MEASURE DHS;* Ghana DHS 2008 Ghana DHS 2014 Kenya DHS 2008 and Kenya DHS 2014 *Cleaning and organization of DHS data:* Dropping of irrelevant variables Merger of data sets *Variable management* recoding and renaming *Preliminary analysis of DHS data (secondary data)* Hypothesis testing Analysis of association and Determination of significant levels (set at ≤ 0.5)	Gaining entrée for primary data collection *Primary data collection* In-depth interviews (IDIs) of service providers Focus Group Discussion (FGD) among pregnant women *Preliminary analysis of qualitative data* Data transcription Annotation of significant statement Coding and Categorization of common themes *Key Informant Interviews* Transcription of key informant interviews *Triangulation of data* Drawing congruence of policy user Perspective/perception, Service provider perspective and Policy implementers view points	*Presentation of preliminary findings and feedback at;* Institutional level Clinical forum ANC clinic to policy users *Summary presentation at;* Regional Annual Performance Review *Presentation of final report at;* School of Public Health, University of Ghana *Publication of Final report for policy actors, research and scientific community in;* MOH reports NHIA report Internal journals Local journals Policy briefs
Monitoring and evaluation of phase I, II, and III		
Guidance and comments of Co-investigator 1,2 and 3	Guidance and comments of Co-investigators 1 and 2	Scheduling of presentation Purview of Co-investigators and faculty of Health Policy Planning Management of the University of Ghana Public Health School

### Data analysis

The secondary data will be cleaned and organized, dropping irrelevant variables and renaming covariates for ease of reference as necessary. Essentially, variables of interest in this study are binary outcomes i.e. 1 = case/the presents of an intervention, and 0 = no case, the absence of intervention, control.

Both multivariate logistic regression and Poisson with robust standard error, or negative binomial regression depending on whether there is over dispersion (conditional variance exceeding the conditional mean) will be fitted and analyzed. Thus, **loge** {*p*1 / *(1—pi)*} = *b0* + *b1 Xi1* + *b2 Xi2* + … + *bkXik*. The Use of the multiple logistic regression model also comes with the advantage of adjusting for confounding by including the covariates in the model.

For the matching method, Ghana’s DHS data sets (2008 and 2014) will be merged. This will increase the sample size to n = 8876, and add power to the study. The merger will also eliminate the risk of lack of common support, the so-called curse of dimensionality, a common phenomenon in propensity score matching, where treated units propensity scores cannot find suitable pairs.

Descriptive data will be presented using tables, frequencies, and proportions. The data will then be analyzed distinctly, using multiple statistical models and quasi-experimental design techniques. Firstly, the student independent *t*-test will be used to test for proportions, the significance of association, and mean differences between the independent variable, and the binary outcomes of interest; antenatal care uptake, and facility delivery utilization.

Test of association set < 0.05 less of significance, between the secondary variables of interest (stillbirth, perinatal death, and neonatal mortality) and the covariates; mothers age, marital status, level of education, wealth index, region, area of residence, twin delivery, parity, and employment status will be determined by using the Rao Scot Chis-square, which has an added sensitivity advantage, while adjusting for complex survey design features (weighting, clustering and stratification).

Only covariates found to be significantly associated with the dependent or independent variables will then be included in a multiple logistic regression model and adjusted for confounding [72].

Logistics regression is considered appropriate, giving the binary status variables of interest; antenatal care up-take [yes = 1 (ANC visit ≥ 4)] and [no = 0 (ANC visit of ≤ 3]; facility delivery utilization [yes = 1 (facility delivery) and no = 0 (home delivery)], and the secondary outcome variables; stillbirth, perinatal and neonatal deaths, generated through the re-coding of the relevant variable *m14, m15, b6, b7*, and *s1015,* from the DHS data sets (Table 5).

The treatment difference will be determined through nearest-neighbor matching of treated and untreated units’ propensity scores generated ex-post, and using logistics regression while controlling for the confounding variables for precision. The 'free' policy impact will then be determined using the average treatment effect on the treated. Robustness of results will be achieved with reduced standard errors using bootstrapping replication while testing for sensitivity using a t-test of bias, and probit regression.

Of the treatment and comparison country analysis, the DID analysis will be used to determine the policy impact, using the 2008 data set of the two countries (Ghana and Kenya) as a baseline and 2014 data sets as the end line. The study is powered by the sample size calculation, originally determined by the survey design. The sample size is n = 2992/5884 (8,876) for Ghana DHS 2008/2014 data sets, and n = 6079/20,964 (27,043) for Kenya DHS 2008/2014 data sets respectively.

Clustering and stratification will be adjusted for using sample survey weighting at all stages of the analysis. This will produce unbiased treatment estimate that can be generalized to the original survey population [26, 52].

The qualitative data will be deductively analyzed using predefined themes. However, common themes that emerges from the interviews and focused discussion will be organized as sub-themes and inductively reported. The approach will be aided by NVIVO 12, through which nodes and their children will be created to categories significant statements into similarities and dissimilarities [73].

The results of the primary data will be reported using the common themes approach with word frequency count and cloud, and vivid quotations to back themes. The inclusion of qualitative data will add context and situate the discussion with explanatory power to the statistical findings [48].

## Discussions

The study design and methodology will be discussed as deliverables outlining the methodological strength and limitations for future researchers. Multistage discussions will be employed adequately and distinctly, in reporting the study findings. The predictive abilities of the variable and the covariates in the multiple logistic regression model will be thoroughly examined along the lines of statistical significance levels, set at p < 0.05. The study discussion will also compare and contrast the current result with exiting literature for congruence or divergence.

The effects of facility delivery utilization i.e. antennal clinic attendance and health facility delivery will be discussed in the context of financial accessibility and universal health coverage. Stillbirth and perinatal deaths outcomes will be modeled to make predictions and projections in the discussion stage and inferred on targets such as achieving sustainable development goal 3.

The discussion will also look at the ‘free’ policy effectiveness and impact, drawing lessons from the qualitative result in the context of lower/middle income country, and the overall objectives of social health insurance in developing country context to inform policy development in other countries, particularly, Sub-Saharan Africa.

In the qualitative stage, discussions will follow the thematic inductive approach, where meanings from participants’ views are compared to existing literature to establish similarities and dissimilarities. The discussions are expected to identify policy implications from the results to inform practice and research. Discussions will isolate key study findings and highlight the contribution of the study to knowledge and policy, i.e. what is known from the study, in an attempt to address the research objectives and gaps.

Conclusions will be drawn for policymakers and recommendations made for policy, practice, and research. Within the study context, the conclusions will take the shape of the data as it is compared to existing literature, mirroring Lipsky's theory of Street-Level Bureaucracy to draw lessons for future design and implementation of a similar intervention in similar settings.

### Expected outcome

This study deliverable will include a report on the study findings, indicating contribution to knowledge, the implication for policy and research, and a recommendation for practice. It is expected that the analysis shows proof of positive policy effect on maternal health care utilization and therefore increasing access to utilization.

A significant level of associations is expected from the primary outcomes as a consequence of the policy effect. This is also expected to show a positive correlation to desirable outcomes of interest; stillbirths, perinatal and neonatal mortalities.

A theory of change model will also be developed as an expected outcome of this study, to help address policy implementation framework, inadequacies, and policy workability gaps. Policy re-examination and policy modification are expected within the context of the study findings.

### Dissemination of findings

This protocol is approved for implementation as independent research by the Principal Investigator. Thus, a final copy will be submitted to the graduate school of the University of Ghana as a requirement for the award of a doctor of philosophy degree in public health. The study results will be shared with participants in the Upper East Region of Ghana, from where the primary data is drawn from, through annual performance review meetings.

The study results will also be shared at antenatal and post-natal clinic schools in brief and synopsis using local dialects to enhance pregnant women's understanding. Extracts from this study will be published in peer-reviewed journals of local and international repute to educate and inform the larger scientific community.

## Supplementary information

**Additional file 1**: Plan for Implementation

## Data Availability

This study is reliant on secondary data sets from MEASURE DHS for Ghana and Kenya available from www.dhsprogram.com/data/. However, public access to this data is restricted and cannot be shared with a third party, unless with a reasonable request and upon the permission of MEASURE DHS.

## Abbreviations

ANC: Antenatal care
CHAG: Christian Health Association of Ghana
CPHS: Community Partnership for Health Service
CZ: Central zone
DD: Difference-in-differences
DPPME: Director, policy planning monitoring and evaluation
EZ: Eastern zone
FGD: Focus group discussion
FMHCP: Free maternal health care policy
GDHS: Ghana demographic and health survey
GHS: Ghana health service
H: Hospital
HC: Health centre
IDI: In-depth interview
ILF: Individual-level factors
KII: Key informant interview
MOH: Ministry of Health
NA: Not applicable
NHIA: National Health Insurance Authority
NHIS: National Health Insurance Scheme
PPME: Policy planning monitoring and evaluation
PNC: Postnatal care
PIS: Participants information sheet
POW: Programme of work
PSM: Propensity score matching
PSU: Primary sampling unit
PW: Probability weighting
RD: Regional director
SLB: Street-level bureaucracy
SPF: Service provider factors
SVY: Survey
UER: Upper east region
UNICEF: United Nations International Children’s Emergency Fund
WHO: World Health Organization
WGT: Weighting
WZ: Western zone
